# Woolf et al’s “GWAS by subtraction” is not useful for cross-generational Mendelian randomization studies

**DOI:** 10.1186/s13104-025-07326-9

**Published:** 2025-06-26

**Authors:** David M Evans, George Davey Smith, Gunn-Helen Moen

**Affiliations:** 1 Institute for Molecular Bioscience, The University of Queensland, Brisbane, Australia; 2 The Frazer Institute, The University of Queensland, Woolloongabba, QLD 4102 Australia; 3 MRC Integrative Epidemiology Unit, University of Bristol, Bristol, UK; 4 Institute of Clinical Medicine, Faculty of Medicine, University of Oslo, Oslo, Norway; 5 Department of Public Health and Nursing, K.G. Jebsen Center for Genetic Epidemiology, NTNU, Norwegian University of Science and Technology, Trondheim, Norway

**Keywords:** Mendelian randomization, Smoking, GWAS by subtraction, Paternal effects, Maternal effects

## Abstract

Mendelian randomization (MR) is an epidemiological method that can be used to strengthen causal inference regarding the relationship between a modifiable environmental exposure and a medically relevant trait and to estimate the magnitude of this relationship [1]. Recently, there has been considerable interest in using MR to examine potential causal relationships between parental phenotypes and outcomes amongst their offspring [2–4] (interestingly one of the earliest exemplars of MR was confirmation that antenatal maternal folate was protective against offspring neural tube defects [1]). In a recent issue of *BMC Research Notes*, Woolf, Sallis, Munafo and Gill (2023) [5] (abbreviated as WSMG from now on) present a method they call “GWAS by subtraction” (not to be confused with GWAS by subtraction via genomic SEM [6, 7]), to derive genome-wide summary statistics for paternal smoking and other “paternal phenotypes” with the goal that these estimates can then be used in downstream (including two sample) MR studies [8]. Whilst a potentially useful goal, WSMG (2023) focus on the wrong parameter of interest for useful genome-wide association studies (GWAS) and downstream cross-generational MR studies, and the estimator that they derive is neither efficient nor appropriate for such use.

The paper is peppered with inaccuracies that make it difficult for readers to make sense of what the authors have done, including errors in equations. Rather than itemizing these- although they require correcting- we restrict ourselves to pointing out some of the issues that are directly relevant to cross-generational genome-wide association studies and the utilization of the SNP-phenotype regression coefficients from these studies in downstream MR analyses. Our letter focuses on the following issues in particular: (1) WSMG’s estimator is not conceptually coherent; (2) WSMG’s estimator is not statistically efficient; (3) WSMG’s formula for the standard error of their estimator is likely to be conservative; (4) WSMG’s empirical analyses use measures of own and maternal smoking that are too different to be combined in their proposed fashion; (5) WSMG’s estimator is unlikely to be useful from the perspective of locus discovery; (6) The empirical MR analyses the authors conduct are equivalent to an (underpowered) MR of own smoking on own lung cancer/emphysema/bronchitis; and (7) WSMG’s estimator is not a useful quantity for estimating the causal effect of paternal phenotypes on offspring outcomes.

1. **WSMG’s estimator of βCG_FP is not conceptually coherent.**

Following WSMG, we define the following population level quantities:

β_CG_FP_ = Population regression coefficient of father’s phenotype on child genotype (the parameter of interest to WSMG).

β_CG_MP_ = Population regression coefficient of mother’s phenotype on child genotype.

β_CG_CP_ = Population regression coefficient of child’s phenotype on child genotype.

We assume for the moment that β_CG_FP_ is a useful quantity to estimate (however, see below on this point). The estimator of β_CG_FP_ that WSMG propose in their paper is:

$$\:{\widehat{\beta\:}}_{CG\_FP}={\widehat{\beta\:}}_{CG\_CP}-{\widehat{\beta\:}}_{CG\_MP}$$

with standard error:

$$\:{\text{s}\text{e}(\widehat{\beta\:}}_{CG\_FP})=\sqrt{\text{v}\text{a}\text{r}\left({\widehat{\beta\:}}_{CG\_CP}\right)+\text{v}\text{a}\text{r}\left({\widehat{\beta\:}}_{CG\_MP}\right)}$$

where $\:{\widehat{\beta\:}}_{CG\_CP}$ is the coefficient from the regression of child phenotype on child genotype in the sample, and $\:{\widehat{\beta\:}}_{CG\_MP}$ is the coefficient from the regression of maternal phenotype on child genotype in the sample.

Since WSMG’s estimator combines regression coefficients from two generations and collapses across sexes, the estimator implicitly assumes no sex differences in genetic effects or differences in genetic effects across generations. We note that these assumptions are unlikely to hold for a phenotype such as smoking. Cigarette smoking behaviour has changed rapidly in many societies, in some having risen from zero to the majority of the population, and then back to a small minority in less than a century. The distribution of smoking by sex, socioeconomic class, age and ethnicity has similarly fluctuated markedly over this time. This could lead to situations in which it is clear that nonsensical estimates would be made using the GWAS by subtraction approach advocated by the authors. Consider a population in which female smoking is near zero (such as in some African, Asian and Middle Eastern countries currently) and the GWAS by subtraction approach was carried out. The process must, of course, be symmetrical- it should be agnostic to the sex of the parent you have phenotypic data for. If you had paternal smoking data you would produce GWAS by subtraction summary statistics for maternal smoking that could then be used in MR studies to produce estimates of the effects of maternal smoking on offspring and partner outcomes. These would suggest positive influences of maternal smoking on their own and their offspring and partner outcomes even in a situation where no mothers smoked. Indeed, these findings would be similar to the ones that the authors of the paper under discussion have reported with respect to the effects of paternal smoking on their own, their offspring and their partner outcomes [5, 8].

(2) **WSMG’s estimator of β**_**CG_FP**_ **is not statistically efficient.**

WSMG’s estimator is not an efficient estimator of β_CG_FP_ since it equally weights contributions from the regression of child’s phenotype on child’s genotype, and mother’s phenotype on child’s genotype. A superior estimator that is appropriate for the analysis of common genetic variants in large samples uses inverse variance weighting (i.e. the most statistically efficient form of weighting a linear combination of estimators) and treats the sample regression coefficient of mother’s phenotype on child genotype as one estimate of β_CG_FP_, and half the sample regression coefficient of own phenotype on own genotype as another i.e.:

$$\:{\widehat{\beta\:}}_{CG\_FP}=\frac{{{w}_{1}\times\:0.5\times\:\widehat{\beta\:}}_{CG\_CP}+{w}_{2}\times\:{\widehat{\beta\:}}_{CG\_MP}}{{w}_{1}+{w}_{2}}$$

where

$$\:{w}_{1}=\frac{4}{\text{v}\text{a}\text{r}\left({\widehat{\beta\:}}_{CG\_CP}\right)}$$

$$\:{w}_{2}=\frac{1}{\text{v}\text{a}\text{r}\left({\widehat{\beta\:}}_{CG\_MP}\right)}$$.

with standard error:

$$\:\text{s}\text{e}\left({\widehat{\beta\:}}_{C{G}_{FP}}\right)=\sqrt{\left(\frac{{.5w}_{1}}{{w}_{1}+{w}_{2}}{)}^{2}\text{v}\text{a}\text{r}\right({\widehat{\beta\:}}_{CG\_CP})+(\frac{{w}_{2}}{{w}_{1}+{w}_{2}}{)}^{2}\text{v}\text{a}\text{r}\left({\widehat{\beta\:}}_{CG\_MP}\right)+\text{c}\text{o}\text{v}({\widehat{\beta\:}}_{CG\_CP},{\widehat{\beta\:}}_{CG\_MP})\frac{{{w}_{1}w}_{2}}{{{(w}_{1}+{w}_{2})}^{2}}}$$

Like WSMG’s estimator, as well as the usual assumptions of no assortative mating, no indirect effects, and no population stratification, this more efficient estimator assumes no difference in genetic effects between males and females, no difference in genetic effects across generations, and that mothers and offspring are measured on the same continuous phenotype on the same measurement scale (or that their measurements can be transformed to the same continuous scale).

(3) **WSMG’s formula for the standard error of their estimator is only correct under independence and therefore likely to be conservative in their own and other real-world applications.**

The formula for the standard error that WSMG uses is only correct under the assumption that $\:{\widehat{\beta\:}}_{CG\_CP}$ and $\:{\widehat{\beta\:}}_{CG\_MP}$ are independent (i.e. estimates of SNP-phenotype association are derived from completely independent samples and/or there exists no residual covariance between maternal phenotype and offspring phenotype over and above the SNP-phenotype associations). Rather, the correct formula for the standard error of their estimator allowing for covariance between the regression coefficients is:

$$\:{\text{s}\text{e}(\widehat{\beta\:}}_{CG\_FP})=\sqrt{\text{v}\text{a}\text{r}\left({\widehat{\beta\:}}_{CG\_CP}\right)+\text{v}\text{a}\text{r}\left({\widehat{\beta\:}}_{CG\_MP}\right)-2\text{c}\text{o}\text{v}({\widehat{\beta\:}}_{CG\_CP},{\widehat{\beta\:}}_{CG\_MP})}$$

We note that, if necessary, the covariance term in the above formula can be estimated by e.g. bivariate LD score regression [9]:

$$\:\widehat{\text{c}\text{o}\text{v}}({\widehat{\beta\:}}_{CG\_CP},{\widehat{\beta\:}}_{CG\_MP})\approx\:\frac{{N}_{S}}{\sqrt{{N}_{CG\_CP}{N}_{CG\_MP}}}\rho\:\sqrt{\text{v}\text{a}\text{r}\left({\widehat{\beta\:}}_{CG\_CP}\right)\text{v}\text{a}\text{r}\left({\widehat{\beta\:}}_{CG\_MP}\right)}$$

$$\:=\widehat{int}\times\:\text{s}\text{e}\left({\widehat{\beta\:}}_{CG\_CP}\right)\text{s}\text{e}\left({\widehat{\beta\:}}_{CG\_MP}\right)$$

where N_CG_CP_ is the number of individuals in the regression of child phenotype on child genotype, N_CG_MP_ is the number of mothers in the regression of maternal phenotype on child genotype, N_S_ is the effective sample overlap between the two regressions, *ρ* is the correlation between maternal and child phenotypes, and $\:\widehat{int}$ is the bivariate LD score regression intercept [10]. Because the residual covariance between maternal and offspring phenotypes is likely to be positive, any sample overlap should result in a smaller standard error than what WSMG proposes. The corollary is, other important considerations aside, that #2 and #3 imply that WSMG’s empirical analyses of smoking in the UK Biobank are likely to be conservative.

(4) **WSMG’s empirical analyses use measures of smoking that are different.**

WSMG’s estimator assumes that offspring and maternal phenotypes have been measured on the same (continuous linear) scale, however, their empirical analyses in the UK Biobank use phenotypes where this assumption is highly questionable (i.e. a GWAS of “lifetime smoking index” versus a maternal GWAS of smoking/non-smoking). It is not appropriate to combine the regression coefficients of different phenotypes in the fashion that WSMG suggest regardless of whether the phenotypes (or the regression coefficients) have been standardized.

(5) **Estimation of**$\:{\widehat{\beta\:}}_{CG\_FP}$ **is unlikely to be useful from the perspective of genetic locus discovery.**

As shown above, WSMG’s estimator is not efficient. Since there is an implicit assumption of no generational effects, far more useful would be an estimate of own genotype-own phenotype association that combines information from the GWAS of own phenotype with information from a GWAS of maternal phenotype. We and others have shown various ways of incorporating parental and offspring GWAS to improve the power of locus discovery [11–14].

(6) **The Empirical MR analysis the authors conduct is equivalent to an (underpowered) MR of own smoking on own lung cancer/emphysema/bronchitis.**

The authors use $\:{\widehat{\beta\:}}_{CG\_FP}$ in downstream MR analyses to investigate whether paternal smoking causes paternal lung cancer/bronchitis/emphysema. However, assuming the absence of generational effects, this analysis is basically the same (albeit a less powerful way) of investigating whether (own) smoking causes (own) lung cancer.

(7) $\:{\widehat{\varvec{\beta\:}}}_{\varvec{C}\varvec{G}\_\varvec{F}\varvec{P}}$ **is not a useful quantity for estimating the causal effect of paternal phenotypes on offspring outcomes.**

A far more interesting empirical question than the one addressed in WSMG involves estimating the causal effect of paternal smoking on offspring phenotypes. Indeed the authors went on to do precisely this in a subsequent preprint using their summary statistics [8]. Unfortunately, the summary statistics derived from WSMG cannot be used for this purpose, which WSMG apparently recognise, since they have withdrawn their preprint which produced clearly ludicrous effect estimates. Rather, for these sorts of analyses, what is required is an estimate of the association between paternal genotype and offspring outcome (conditional on offspring genotype). These estimates could be obtained by e.g. conditional analysis of genotyped father-offspring pairs/parent-offspring trios, and then used in one or two sample MR studies. Indeed, similar investigations have been proposed (and performed) in MR studies of maternal exposures and offspring outcomes [2, 4, 15]. In other words, “conditional GWAS” rather than “GWAS by subtraction” is required for valid cross-generational MR studies.

Alternatively, an MR by proxy design could be used to investigate the causal effects of parental exposures on offspring outcomes, in which offspring genotype proxies for maternal (or paternal) genotype [16]. However, in order for this sort of design to be informative for causality, it requires data on the parental phenotype and an informative genotype by environment interaction. Indeed, even if these prerequisites are satisfied, there may still be limitations with respect to which parental exposure-offspring phenotypes can be validly examined for causality. For example, the first study to employ this design [16], used a SNP in the offspring *CHRNA5* gene (i.e. a genetic variant related to smoking) to proxy maternal genotype at the same locus, and investigate the relationship between maternal smoking during pregnancy and offspring birthweight. The authors reasoned that any association between offspring SNP and offspring birthweight due to a causal effect of maternal smoking on offspring birthweight, would only be observed in offspring whose mothers smoked during pregnancy. Indeed, it was critical that maternal smoking status during pregnancy was available in order for the authors’ design to yield valid causal inferences. However, the same design would have less validity to examine the effect of maternal smoking during pregnancy and e.g. later life outcomes as such an association could be generated through post-natal maternal smoking and/or offspring smoking [17].

Finally, we note that the authors have failed to mention several recent methodological extensions of genetic association studies that permit estimation of maternal, paternal and offspring genetic effects when parent-child duos/trios are not readily available, and that can be used in downstream cross-generational MR studies [2, 10, 18–23]. These includes approaches that statistically impute parental genotypes using related individuals’ genotypes in situations where parents have not been physically genotyped [21–23]. There is a century old literature from animal and human genetics that discusses the value of relatives with genotypes but no phenotype, or with phenotypes but no genotypes [24], but the notion of strengthening causal inference through simply thinking of a parental group - with neither phenotype nor genotype data available for them (whether imputed or otherwise)– is alchemic. Extensive discussion of cross-generational MR methodology as well as some recent developments are available elsewhere [2, 3].

## Data Availability

Not applicable.

## Abbreviations

GWAS: Genome-wide association study
LD: Linkage disequilibrium
MR: Mendelian randomization
